# Hashimoto’s Thyroiditis Presenting As Non-specific Low Back Pain: A Case Report on Diagnostic Challenges and Management in Primary Care

**DOI:** 10.7759/cureus.58084

**Published:** 2024-04-11

**Authors:** David R Annison, Afsaneh Abedi, Michael Mansfield

**Affiliations:** 1 Orthopaedics, Academic Centre for Surgery, South Tees Hospitals NHS Foundation Trust, Middlesbrough, GBR; 2 Physical Medicine and Rehabilitation, Pure Physiotherapy-Greenfield Surgery, Nottingham, GBR; 3 Physical Medicine and Rehabilitation, School of Sport, Exercise, and Rehabilitation Sciences, University of Birmingham, Birmingham, GBR

**Keywords:** autoimmune hypothyroidism, : musculoskeletal pain, thyroid-stimulating hormone, hashimoto thyroiditis, low back pain

## Abstract

Non-specific low back pain (NSLBP) may account for 90-95% of cases of low back pain presenting to primary care. Clinicians should remain vigilant however to non-spinal musculoskeletal conditions that may mimic NSLBP and musculoskeletal complaints.

We present a case of a 38-year-old female with low back pain, lower limb tightness, groin pain, and leg cramps. Symptoms failed to improve with physiotherapy and subsequent blood tests revealed elevated thyroid-stimulating hormone (TSH), and elevated thyroid peroxidase antibody (TPO). The patient was diagnosed with hypothyroidism secondary to Hashimoto's thyroiditis (HT), an autoimmune endocrine thyroid disorder. Levothyroxine 100 microgram(µg) was prescribed, and clinical symptoms improved within eight weeks.

Clinicians may wish to consider thyroid dysfunction when patients with common musculoskeletal complaints, weight gain, and fatigue respond atypically to evidence-based physiotherapy management.

## Introduction

Non-specific low back pain (NSLBP) is estimated to affect around 619 million people worldwide and is the leading cause of years lived with disability in 126 countries [1]. NSLBP pain constitutes 90%-95% of low back consultations in primary care and is a condition commonly assessed and managed by Physiotherapists [1, 2]

Hashimoto’s thyroiditis (HT), an autoimmune endocrine thyroid disorder, can masquerade as NSLBP and other non-specific musculoskeletal (MSK) conditions [3, 4]. Affecting females more than males, at a ratio of 12:1, HT has greater prevalence in the 40-60 age group, and if untreated, is a common cause of hypothyroid disease [3]. Individuals with hypothyroid disease can present with constipation, fatigue, weight loss, and muscle and joint pain [3].

Although the exact aetiology of this condition is unknown; genetic, and environmental factors are thought to play a role in the development of HT. Factors include radiation exposure, excessive iodine levels, gastrointestinal bacterial infections, and individuals with a family history of autoimmune and thyroid conditions, including inherited genes, *HLA-DR*3 and *HLA-DR5 *[4, 5].

Individuals with HT are initially asymptomatic but can progress to develop MSK manifestations such as joint stiffness and muscle weakness [4, 6, 7]. Severe or unmanaged hypothyroidism can cause significant proximal myopathy [6].

An association between hypothyroid dysfunction and MSK conditions such as adhesive capsulitis, Dupuytren’s contracture, trigger finger, and carpal tunnel syndrome has been reported [4, 7, 8].

Variations in clinical manifestations can make diagnosis challenging. Case reports of Hashimoto’s thyroiditis have been published within Neurology, Orthopaedics, and other subspecialty fields of medicine. Within musculoskeletal caseloads, differential diagnoses of connective tissue disorders may be considered [9]. 

This case report discusses the identification, and management of HT in a 38-years of age female presenting to a physiotherapy clinic in primary care with low back pain.

## Case presentation

A 38-year-old female was referred to an outpatient musculoskeletal (MSK) physiotherapy clinic with a five-month subjective history of low back pain and lower extremity muscle stiffness. Precipitated by a ‘jolt’ whilst walking the dog, the patient denied any prior lower back or pelvic problems. No loss of appetite, abdominal pain, or irregular menstrual cycle was reported. A weight gain of 4-5 kilograms (kg) was self-reported without dietary or activity change. The patient reported no physical hobbies or activities and a sedentary job.

**Figure 1:**
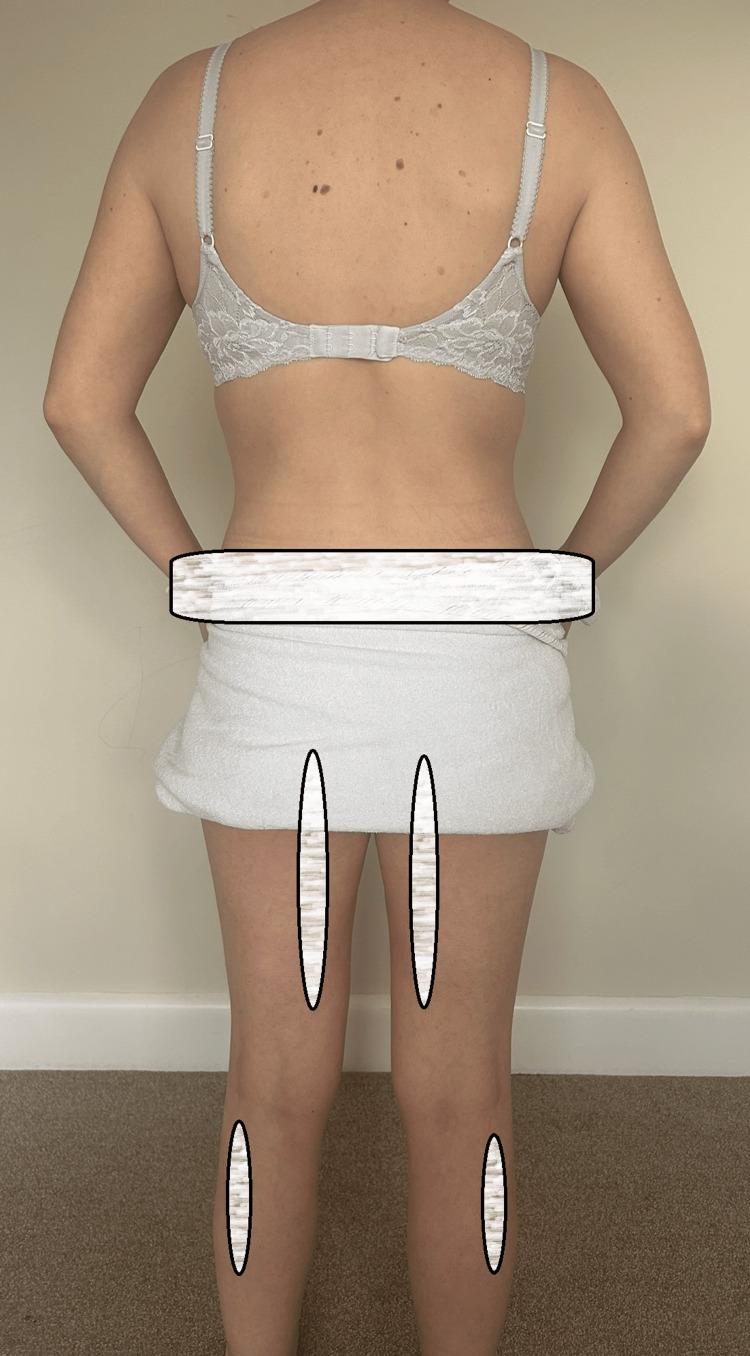
Areas of discomfort superimposed on the patient's photo

Pain in the lumbar spine region was described as dull in nature with a numeric rating scale (NRS) of 4 out of 10 at rest. Progressively worsening bilateral posterior thigh and lower leg aching was reported. Deep dull aching from the groin to the distal inner thigh bilaterally was also noted (Figure 1).

Walking and computer work for twenty and forty minutes, respectively, aggravated her symptoms. No paraesthesia or anaesthesia in the lower limbs was reported nor were bladder or bowel symptoms on questioning.

The patient denied any family history of inflammatory conditions, thyroid disease, cancer, fibromyalgia, or chronic fatigue. A self-initiated two-week trial of non-steroidal anti-inflammatories was reported as ineffective and the patient was advised intermittently utilising paracetamol to manage symptoms. The patient was taking magnesium as a supplement and had recently ceased an ineffective trial of Amoxicillin, prescribed for suspected cystitis.

Lower back and hip mobility were considered within the normal range for age and lifestyle. Symmetrical global tenderness on gross palpation to the lower back, groin, hamstring, and lower legs was noted. Assessment of upper and lower limb dermatomes, myotomes, and reflexes was unremarkable. The examination was in keeping with non-specific low back pain and non-radicular referral of pain [2]. No escalation of care or further imaging was required or recommended at presentation due to the absence of concerning features [2, 10].

A 12-week period of education, self-management advice, structured exercise, and simple analgesia, as recommended by the World Health Organisation, was provided. Despite treatment, symptoms failed to improve, and the case was discussed with the onsite medical general practitioner (GP). Standard blood tests were requested as a first-line investigation and the results are presented in Table 1. 

**Table 1:** Pertinent blood results High ↑; Low ↓ TSH: thyroid stimulating hormone; ANA: anti-nuclear antibodies; CRP: C-reactive proteins; abs: antibodies

Test	Patient Result	Normal Range	Units
Creatinine	↑ 97	45-84	umol/L
Cholesterol	↑ 6.13	0-5.0	mmol/L
Triglycerides	↑ 2.75	<1.7(Fasting)	mmol/L
TSH	↑ >100	0.27- 4.20	Mu/L
T4 Total	↓ 41.7	66-181	nmol/L
Free T5	↓ 4.7	12.0- 22.0	pmol/L
Free T4	↓ 2.32	3.1-6.8	pmol/L
Anti-Thyroid peroxidase abs	↑ 88	<34	IU/Ml
Anti-Thyroid peroxidase abs	↑ 85.6	<34	IU/Ml
Anti-Thyroglobulin Abs	54	<115	IU/Ml
ANA	Negative		
Vitamin D (25 OH)	55	Adequate 50-<75	nmol/L
Vit B12	235	145- 569	pmol/L
CRP	3.91	<5.0	mg/L

Laboratory results (Table 1) revealed elevated Thyroid stimulating hormone (TSH) >100 mU/L (normal reference ranges 0.27- 4.20 mU/L) indicating hypothyroidism. Thyroid peroxidase antibody (Anti-TPO) was elevated at 88 IU/M, (normal reference range < 34 IU/mL), and an abnormal lipid profile was highlighted (cholesterol level; 6.13 mmoI/L, triglycerides; 2.75 mmoI/L, Non-high-density lipoprotein (HDL) cholesterol; 4.18 mmoI/L). 

The diagnosis of hypothyroidism secondary to Hashimoto's thyroiditis was confirmed by a general practitioner, and a six-week daily dose of 100 micrograms of levothyroxine was prescribed. Symptoms remained unchanged for the first three weeks following medication commencement and the patient was appropriately counselled. At four weeks, the patient reported improved mood and motivation, and a notable reduction in global muscle and joint stiffness. Low back pain was rated as two on the NRS. Blood tests were repeated at six weeks and dosing was subsequently increased to 125 micrograms daily. Two months post medication commencement and the patient no longer required analgesia. Low back pain and lower limb symptoms resolved, and the patient was discharged from physiotherapy. Unfortunately, subsequent thyroid function tests are not available as the clinician relocated.

## Discussion

We report a case of undiagnosed Hashimoto’s thyroiditis presenting as low back pain and lower limb myalgia in a 38-year-old female.

HT is chronic inflammation of the thyroid and is both the most common autoimmune disease and cause of hypothyroid dysfunction [3]. Diagnosis of HT is established based on a range of clinical signs and symptoms and the presence of serum thyroid antibodies, thyroperoxidase, and thyroglobulin [3]. Ultrasound examination is the most common imaging modality in patients with thyroid disease, with its utility reported for both diagnostic and interventional purposes [11].

Musculoskeletal disorders often accompany thyroid dysfunction, but they are also observed in patients with subclinical hypothyroidism [4]. According to thyroid status, Cakir et al. placed 137 patients into two groups and conducted neurological and musculoskeletal assessments. Adhesive capsulitis was found in 10·9% (n = 15), carpal tunnel syndrome in 9·5% (n = 13), and Dupuytren’s contracture in 8·8% (n = 12). The prevalence of adhesive capsulitis was highest in patients with subclinical thyrotoxicosis (17·4%). Despite screening for alternative causes of musculoskeletal symptoms; pain, weakness, and stiffness were commonly reported in those with hypothyroidism [4]. Although the patient within this case report did not present with the specified conditions noted by Cakir, et al., the primary complaints included muscle and joint pain, weakness, and stiffness. 

In our case report, myalgia, muscular cramps, lower back stiffness, and symmetrical lower limb subjective weakness were reported. An observational study conducted by Rodolico et al. explored the relationship between thyroid dysfunction and muscular disturbances, specifically the occurrence of myopathy as the primary clinical presentation of hypothyroidism [12]. All 10 patients who were referred to the neurology department for various muscular symptoms, such as fatigability, myalgia, cramps, and proximal weakness were found to have hypothyroidism attributed to Hashimoto’s thyroiditis [12]. 

Muscle weakness may occur as dysfunction of the nerve-mediated action of thyroid hormones on muscle fibres but myopathy presentations must be confirmed by further investigation [6,13]. Individuals with abnormal thyroid function can suffer from fatigue, poor sleep quality, and muscle pain. Muscle pain and exercise intolerance may be attributable to impaired mitochondrial function, reduced adenosine triphosphate (ATP) production, and low muscle carnitine levels [14].

Auto-immune diseases such as rheumatoid arthritis and systemic lupus erythematous may also present similarly [9]. Screening for hypothyroidism, with assessment of thyroid stimulating hormone (TSH) and thyroid antibody is therefore recommended and reflects the elevated levels noted in this case [9]. Thyroglobulin antibody (Tg-Ab) levels were however normal, which again reflects the lack of sensitivity of Tg-Ab compared to thyroid peroxidase antibodies reported in the literature [15]. The National Institute for Health and Care Excellence (NICE) has produced the NG145 guideline to provide guidance in the assessment and management of thyroid disease [16]. NG145 recommends thyroxin replacement for primary hypothyroidism, at 1.6 micrograms of levothyroxine sodium per kilogram of body weight per day for adults under 65 [16]. The British National Formulary further supports the NG145 recommendations and advises that the TSH level should be assessed every three months until the TSH level is stabilised [17].

## Conclusions

In this case report, low back pain and bilateral lower limb myalgia symptoms resolved eight weeks following levothyroxine treatment for Hashimoto's thyroiditis. The report aims to raise awareness of the signs and symptoms of hypothyroid disease that can masquerade as common musculoskeletal complaints. 
